# NAC4ED: A high‐throughput computational platform for the rational design of enzyme activity and substrate selectivity

**DOI:** 10.1002/mlf2.12154

**Published:** 2024-12-25

**Authors:** Chuanxi Zhang, Yinghui Feng, Yiting Zhu, Lei Gong, Hao Wei, Lujia Zhang

**Affiliations:** ^1^ Shanghai Engineering Research Center of Molecular Therapeutics & New Drug Development School of Chemistry and Molecular Engineering, East China Normal University Shanghai China; ^2^ Department of Micro/Nano Electronics School of Electronic Information and Electrical Engineering, Shanghai Jiao Tong University Shanghai China; ^3^ School of Biotechnology East China University of Science and Technology Shanghai China; ^4^ School of Biotechnology Tianjin University of Science and Technology Tianjin China; ^5^ NYU‐ECNU Center for Computational Chemistry at NYU Shanghai Shanghai China

**Keywords:** high‐throughput screening, near‐attack conformation, protein engineering, rational design

## Abstract

In silico computational methods have been widely utilized to study enzyme catalytic mechanisms and design enzyme performance, including molecular docking, molecular dynamics, quantum mechanics, and multiscale QM/MM approaches. However, the manual operation associated with these methods poses challenges for simulating enzymes and enzyme variants in a high‐throughput manner. We developed the NAC4ED, a high‐throughput enzyme mutagenesis computational platform based on the “near‐attack conformation” design strategy for enzyme catalysis substrates. This platform circumvents the complex calculations involved in transition‐state searching by representing enzyme catalytic mechanisms with parameters derived from near‐attack conformations. NAC4ED enables the automated, high‐throughput, and systematic computation of enzyme mutants, including protein model construction, complex structure acquisition, molecular dynamics simulation, and analysis of active conformation populations. Validation of the accuracy of NAC4ED demonstrated a prediction accuracy of 92.5% for 40 mutations, showing strong consistency between the computational predictions and experimental results. The time required for automated determination of a single enzyme mutant using NAC4ED is 1/764th of that needed for experimental methods. This has significantly enhanced the efficiency of predicting enzyme mutations, leading to revolutionary breakthroughs in improving the performance of high‐throughput screening of enzyme variants. NAC4ED facilitates the efficient generation of a large amount of annotated data, providing high‐quality data for statistical modeling and machine learning. NAC4ED is currently available at http://lujialab.org.cn/software/.

## Impact statement

NAC4ED is an efficient platform for enzyme mutation screening, incorporating four key modules: mutation, docking, dynamics simulation, and evaluation analysis. By employing a “near‐attack conformation” design strategy, NAC4ED accurately characterizes enzyme mutation profiles and substrate adaptability, significantly reducing computational costs while maintaining high precision. This approach offers a streamlined, effective solution for optimizing enzyme‐substrate interactions, advancing the field of computational enzyme engineering.

## INTRODUCTION

As extraordinary biocatalysts, enzymes exhibit high chemical, spatial, and substrate/product selectivity, significantly enhanced reaction rates, and efficiently complete catalytic reactions^1^, ^2^. However, naturally occurring enzyme molecules cannot directly meet the stringent specific enzymatic performance requirements for production, such as temperature tolerance, pH tolerance, catalytic activity, and spatial and chiral selectivity. Therefore, high‐performance engineered enzymes have emerged, and even mutant enzymes that catalyze nonnatural reactions have been custom‐designed^3^, ^4^. Arnold and her colleagues pioneered the first‐generation natural enzyme engineering technique‐directed evolution technology^5^, by integrating efficient screening techniques, such as error‐prone PCR^6^, DNA shuffling^7^, and saturation mutagenesis. The mutation‐screening iterative and stepwise evolution strategy enables the exploration and manipulation of active site catalytic environments by cycling through iterative mutations. The molecular basis of enzyme engineering involves the replacement of one or more amino acids in the protein sequence with 19 other basic amino acids, thereby altering the enzyme's sequence, structure, and function. For an enzyme containing N amino acids, the single‐point saturation mutagenesis space was 19 × N, and the space for X‐point combination mutations was as high as $C_{n}^{x}\times 19X$. The mutation library obtained by directed evolution represents only a small fraction of all possible mutations, and the vast majority are ineffective mutation points, with screening efficiency highly dependent on the enzyme sequence and target substrate^8^. Therefore, a rational design guided by structural and mechanistic understanding is considered a more efficient approach^9^.

Drawing on structural analysis and mechanistic understanding of catalytic reactions, the rapid screening of potential mutants that meet target performance criteria by calculating changes in key parameters before and after mutation has shifted costly “wet lab” research to computer‐driven “dry experiments”, achieving efficient in silico design. Termed as the second‐generation rational design technique for natural enzymes, this approach utilizes molecular docking, molecular mechanics^10^, quantum mechanics, and multiscale molecular simulations to guide the selection of function‐enhancing mutations, which are widely employed for the rapid screening of enzyme variants^11^, ^12^. In particular, the QM/MM technique developed by Karplus et al. effectively addresses the challenge of employing quantum chemical methods for accurately computing reaction processes based on first principles in biological catalytic systems, laying the groundwork for ab initio enzyme studies^13^. However, because of the large number of atoms in enzymes and the complexity of dynamic catalytic processes, high‐precision quantum chemical reaction calculations based on first principles require extensive computational resources, posing significant challenges for predicting and analyzing mutational points. The concept of “near‐attack conformation (NAC)”, initially proposed by Bruice in small molecule systems and later extended to enzyme studies^14^, ^15^, ^16^, is considered to effectively circumvent the contradiction between limited computational resources and the near‐infinite computational demands of complex potential energy surfaces at the full atomic level with femtosecond precision in enzyme catalytic reactions. In the “NAC” theory, favorable conformations for reaction occurrence are inferred from the structures of all Michaelis complexes based on their similarity to the transition states, and the activity can be analyzed through the population of active conformational states. Table 1 systematically summarizes the research conducted in the past 5 years on assessing activity or selectivity based on “NAC”. However, most of these studies have primarily focused on exploring enzyme catalytic mechanisms^28^, ^29^, rather than as design strategies for enhancing enzyme properties. Xu and his colleagues were the first in 2010 to establish the “NAC” of the tetrahedral reaction intermediate of LipK107, thereby determining two enantiomeric binding modes of the intermediate, successfully predicting the enantioselectivity of LipK107 in catalyzing the external racemization of 1‐phenylethanol^30^. Enzyme prediction and design based on “NAC” have gradually evolved since then. Subsequently, Shi and his colleagues employed the theory of “NAC” to construct active conformations of substrates and covalent intermediates with thioesterases and investigated the ring‐closing ability of 12‐ and 14‐membered lactones by soraphenone thioesterase^31^. Although the calculations based on “NAC” mentioned above maintain high precision while avoiding the problem of excessively complex calculations of reaction transition states with ultrahigh degrees of freedom, a systematic description of how to design based on “NAC” and computationally explore the compatibility map of enzyme amino acid mutations with substrates to accurately guide the process of natural enzyme remodeling is currently lacking.

**Table 1:** Near‐attack conformation (NAC) parameters of the enzyme research conducted in the past 5 years.

Enzyme	NAC parameters	Ref.
*Spl* DnaX intein enzyme	D_O_T66‐N,_ D_N_H135‐O_N136_	[17]
DNMT3A‐3 L	D_CSAM‐dC_	[18]
Istidine kinase HK853	D_O_T264‐F_	[19]
Acetylcholinesterase	D_O_S203‐P_	[20]
CYP105AS1	D_O_HEM‐H,_ A_FE‐HEM‐OH_	[21]
HssAChE	D_O_S203‐P,_ A_O_S203‐P‐O_	[22]
Peptidylarginine deiminase 2	D_S_C647‐C_	[23]
ω‑Transaminase	D_NK285‐H,_ A_H‐N‐H_K285_	[24]
Candida antarctica lipase B	D_N_H224‐H_	[25]
NiHyuC	D_S_C171‐C,_ A_S_C171‐C‐O_	[26]
Pullulanases	D_O_D619‐C,_ A_O_D619‐C‐O_	[27]

Here, we propose a natural enzyme design strategy based on “NAC”. Through precise analysis of the physicochemical basis of catalytic reactions, we identify the active conformations that control reaction performance, construct quantitative core models to control specific performance, and combine catalytic distance or free energy for rational design. This allows for rapid screening of enzyme mutants that meet specific functional requirements. We screened the adaptive landscape of mutations during the enzyme design process and streamlined the complex catalytic process into parameters based on the near‐attack model. By applying the design concept of reducing complexity to simplicity, we developed a high‐performance enzyme mutant design platform, NAC4ED (NAC for enzyme design). This platform leverages near‐attack states to enhance enzyme design and can be effectively used throughout the enzyme design process. The platform aims to achieve high‐throughput automated design and screening of mutants for specific enzyme reactions, thereby enhancing screening efficiency. NAC4ED, based on an understanding of the specific enzyme‐substrate binding mechanism, allows users to rapidly construct “NAC” catalytic models through molecular docking and perform high‐throughput mutant screening. The platform evaluates effects based on the population and energy of the enzyme‐substrate complex conformations. NAC4ED consists of four modules: mutation, docking, dynamics simulation, and evaluation analysis. NAC4ED is the first enzyme mutation platform designed for high throughput based on the “NAC” model. We anticipate that this software will accelerate the optimization of enzyme design, enabling precise and cost‐effective screening of biocatalysts that meet target performance requirements.

## RESULTS AND DISCUSSION

### NAC4ED design strategy

**Figure 1:**
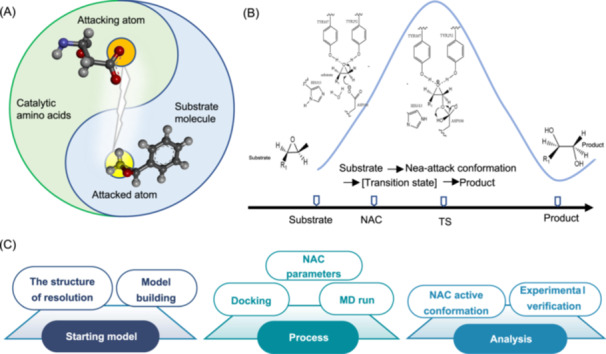
Design strategies based on the “near‐attack conformation (NAC)”. (A) “NAC” parameter model, where orange represents the attacking atom of the key amino acid of the enzyme, and yellow represents the attacked atom of the ligand. (B) Enzyme‐catalyzed states, from complexes to NACs to transition states to products. (C) Steps in the analysis of the “NAC” of an enzyme consisting of three main phases: modeling, processing, and analysis.

Enzyme catalysis involves a multistep reaction, the key of which is the attack of enzyme‐catalyzed amino acids on the substrate after binding, leading to the formation of intermediates that lower the activation energy required for the reaction^32^, ^33^. Different enzymes have different catalytic pathways. However, regardless of the mechanism, the first step—the formation of the enzyme's “near‐attack state”, which stabilizes the transition state of the substrate, is essential for transformation to occur. Therefore, according to the “NAC” theory, all accessible conformations within the *k*_*B*_ *T* level of the lowest energy conformation before the reaction occurs (where *k*_*B*_ is the Boltzmann constant) are categorized into active and inactive conformations. A conformation is considered active if the contact distance between the two atoms that are about to form a new chemical bond is less than the sum of their van der Waals radii, and the bond angle is similar to that of the transition state. These are termed NACs. Thus, the distance or bond angle between two atoms is used as a parameter in the model for the enzyme reaction, termed the enzymes “NAC” model, which can be established (Figure 1A). Based on this, the NAC4ED design strategy was used to construct active conformation based on the catalytic reaction mechanism. These conformations significantly lower the activation energy and stabilize the transition state, thereby capturing the energy required for substrate recognition and specificity (Figure 1B). After obtaining key conformational parameters combined with molecular dynamics simulations, conformational changes over a certain period were analyzed to determine the proportion of active conformations within that timeframe (Figure 1C). This was performed by analyzing the population of active conformations to evaluate the mutagenic effect using Eq. (1).

(1) $$P=\frac{N_{0\left(\mathrm{active}\right)}}{N_{0\left(\mathrm{active}\right)}+N_{1\left(\mathrm{inactive}\right)}}$$

**Figure 2:**
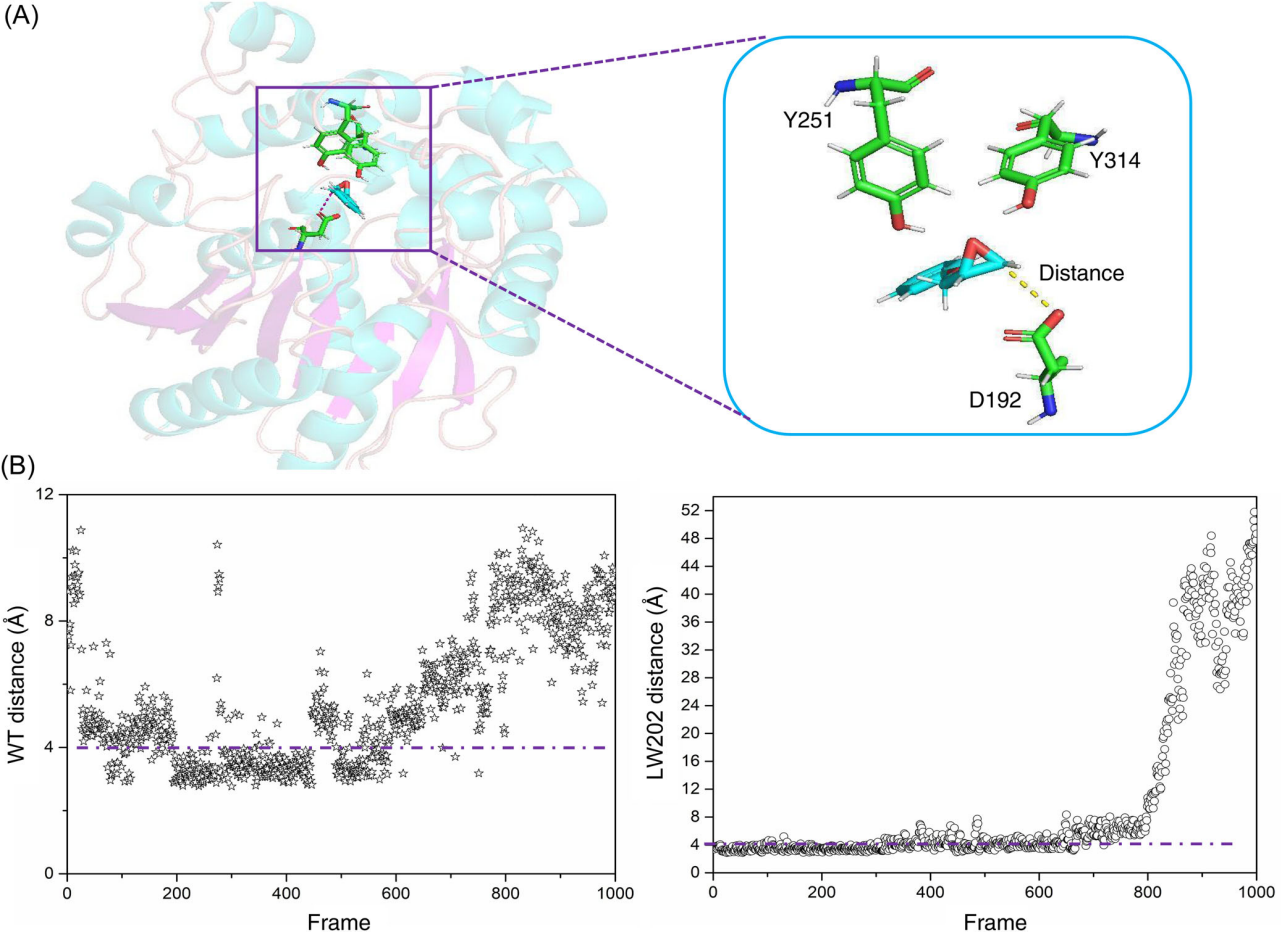
Calculation of the number of active conformations. (A) The NAC conformation of ANEH. D192 is parameterized by the distance between C and styrene oxide. (B) 1000 frames of WT (left) and LW202 (right) calculated as the number of active conformations, with a straight line following the number of frames of active conformations.

To validate the feasibility of the underlying design strategy for NAC4ED, epoxide hydrolases (EHs) were selected as research subjects. EHs (EC 3.3.2.3) catalyze the kinetic resolution of racemic epoxides or the desymmetrization of meso‐epoxides, leading to enantiomer enrichment or production of chiral diols. Reetz et al. obtained a promising variant, LW202, capable of enhancing enantiomeric selectivity in the reaction of rac‐1 using CASTing technology. Despite this, approximately 20,000 clones have been screened^34^. Research on the EHs from *Aspergillus niger* (ANEH) catalytic reaction suggests that D192 initiates a rate‐determining nucleophilic attack on the less hindered C atom^35^, resulting in ring opening and the formation of a covalently bound ester intermediate. Therefore, to rapidly obtain highly adaptive mutations, we employed the NAC4ED design strategy to construct a near‐attack active conformation model for ANEH. The close contact between the attacking O atom of the amino acid residue D192 of ANEH and the C atom of the epoxide undergoing the SN2 reaction is a critical steady state for the reaction to occur^36^. Therefore, the parameter d for defining the active conformation was set as the distance between OD2 of D192 and C2 of the substrate being less than 4 Å, while also requiring interactions between Y314, Y251, and the epoxide group (Figure 2A). After successfully defining the active conformation model, populations of active conformations in the wild‐type (WT) and LW202 Michaelis complexes were compared through molecular dynamics simulations. Averaged over three 50 ns molecular dynamics trajectory analyses, the populations of active conformations for WT and LW202 were determined to be 32.9% and 45.3%, respectively (Figure 2B). This trend is consistent with the results obtained by Reetz^34^, indicating that the establishment of an “NAC” model is conducive to the selection of mutants and demonstrates the feasibility of the NAC4ED design strategy. This study proposes high‐precision three‐dimensional protein structures, deducing stable near‐attack active conformations based on the binding catalytic mechanism of enzymes and substrates, establishing active conformation model parameters, and using this conformation as a starting point for the design. Molecular dynamics simulation conditions were set to test the stability changes in near‐attack states with amino acid mutations and substrate variations. Finally, the population of active conformations in the trajectory was used as a correlation coefficient for mutant evaluation, thereby establishing an efficient enzyme mutation screening strategy based on “NAC”.

### NAC4ED software platform

**Figure 3:**
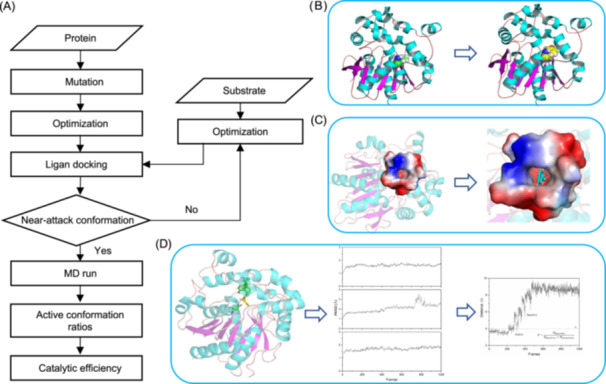
(A) NAC4ED calculation workflow. First, the protein and substrate undergo optimization. The protein is then mutated to generate various mutants, which are structurally optimized as well. (B) The structural changes in the protein before and after mutation. The left side displays the original protein structure, while the right side shows the 3D model of the mutated protein, highlighting changes in key residues. (C) Molecular docking simulations conducted to model the interaction between the protein mutants and the substrate. If the docking results meet the criteria for a NAC, the process proceeds to molecular dynamics (MD) simulations. (D) MD simulation module. Through MD simulations, the active conformation ratio of the mutants over time is analyzed, and their catalytic efficiency is calculated.

Based on this design strategy, we developed the NAC4ED software platform to achieve high‐throughput screening of enzyme mutations with high adaptability. The software framework can be divided into four operational modules, organized in a hierarchical structure from top to bottom, consisting of an amino acid mutation module, a protein‐substrate docking module, a molecular dynamics simulation module, and an evaluation analysis module (Figure 3A). First, the protein‐amino acid module requires a computational model of the enzyme structure, and its modeling quality is evaluated using the structural validation server SAVES (Figure S2). For protein structures that have been resolved, the protein structure can be obtained from ProteinDataBank^37^. However, a large number of protein structures are still unsolved. We obtained the WT protein structure through modeling methods, such as AlphaFold2^38^ and RoseTTAFold^39^. New enzyme mutants were generated based on this original model, in which mutations in amino acids lead to changes in the side‐chain type and conformation. After generating new enzyme mutants, the conformation optimization module for mutants employs molecular dynamics for rapid side‐chain optimization reactions, accurately describing the effects of enzyme conformational changes on substrate binding states (Figure 3B). Second, the protein‐substrate docking module emphasizes whether the pockets and amino acid side chains of the enzyme mutants can geometrically match the substrate molecules after conformational changes. The protocol used in this module is consistent with Reactive Docking^40^, allowing for efficient screening of ligands. By using the designed near‐attack conformational structure parameters to determine the binding state between the substrate and the enzyme mutant, selecting complex states close to the transition state intermediate from different conformations as the starting point for design accurately characterizes the interactions between enzyme mutants and substrate reaction states. Specifically, for different enzyme‐substrate complex systems, we first analyzed the catalytic mechanism to identify the rate‐determining step in the catalytic process. We then calculated the contact distance between the two atoms involved in forming a new chemical bond, based on the key amino acids in this step and the attacked atoms of the substrate molecule. This distance parameter is set to be less than the sum of their van der Waals radii and should also have a bond angle similar to that of the transition state (Figure 3C). Then, the molecular dynamics simulation module emphasizes simulating the stability of enzyme–substrate complexes in the nearest NACs to the transition state in a real environment, obtaining intuitive molecular dynamics trajectories, and statistically sampling the conformational accessible space of the substrate‐enzyme complex to provide a data foundation for efficiently obtaining statistically significant active structure populations (Figure 3D). Finally, the evaluation analysis module calculated the population of active conformations for enzyme‐substrate complex structures, selecting optimal amino acid mutation sites for a specific substrate.

### Efficient screening of EH mutations

**Figure 4:**
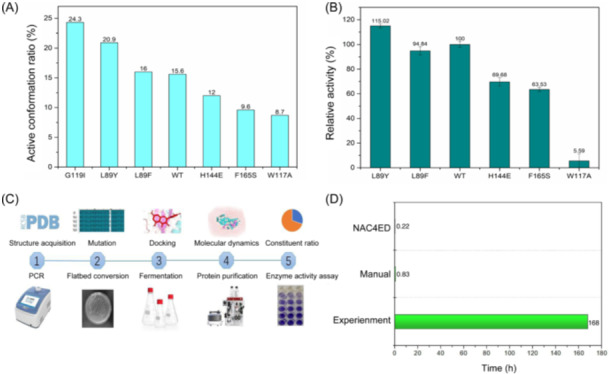
Accuracy and efficiency validation of NAC4ED. (A) Calculation of the active conformation ratio of epoxide hydrolases from *Trichoderma reesei* (TrEH). (B) Determination of the catalytic activity of TrEH mutants. (C) NAC4ED process and experimental steps. (D) Time consumption of NAC4ED automation, manual operation, and experiment.

With the support of the NAC4ED platform, we continued our study using the EHs as the research target. However, in this study, we aimed to perform high‐throughput screening for unknown mutations followed by experimental validation to obtain new high‐adaptability mutants. This was performed to evaluate the high‐throughput screening capabilities of the NAC4ED platform and the adaptability of the selected mutations. To achieve this, we selected the EHs from *Trichoderma reesei* (TrEH), which shares similar mechanisms with many ANEH^41^. Using the NAC4ED platform, an automatic near‐attack active enzyme conformational model was established for TrEH. Mutations were generated and analyzed for changes in the population of active conformations post‐mutation, reflecting the binding status of each variant with the substrate. The high‐throughput screening workflow for TrEH involved several steps. First, the mutation generation module was used to generate variants automatically based on the input TrEH crystal structure (PDB ID: 5uro). Mutations were made in 97 amino acids within 12 angstroms of the bound substrate, leading to a mutation space comprising 1843 variants. During this process, the crystallographic reagents were removed and the protein residue side chains were protonated. Notably, the catalytic amino acid residue D116 of TrEH directly participates in the reaction, whereas Y167 and Y252 anchor to the substrate. Hence, no mutations occurred in these residues. After each variant was generated, energy minimization was performed to ensure that the enzyme remained in a stable conformation after mutation. Based on the catalytic mechanism of the enzyme, the NAC4ED platform was used to establish the near‐attack active conformational models. Substrate structures were imported and subjected to conformational optimization using LigPrep^42^. Each optimal conformation obtained from the previous step was inputted, and a docking box was set around the catalytic active site amino acid D116, with a default distance of 20 Å. The Glide program was used to dock each mutation to the substrate^43^, generating a maximum of 32 docking poses. Notably, not every mutation generated the maximum number of complexes, reflecting the influence of substrate molecules on different spatial hindrances and interactions. After docking, active conformational models consistent with the catalytic mechanism were established, where the distance between the oxygen atom of D116 and the substrate epoxy group's carbon atom was less than 4 Å. Subsequently, molecular dynamics (MD) simulations were conducted for 500 ps and the population of active conformations was calculated using Eq. (1). The results showed that G119I had the highest proportion of active conformations at 24.3%, followed by L89Y at 20.9%, and the WT had an active population of 15.6% (Figure 4A). Experimental validation was conducted for the enzyme mutants, resulting in a relative high activity of 115.02% for the L89Y mutant, which was consistent with the screening results or NAC4ED (Figure 4B). However, despite G119I having the highest proportion of active conformations, its expression performance in experiments was unsatisfactory, and enzyme activity could not be determined. This indicates that although NAC4ED can be used for in silico enzyme activity screening, its expression performance cannot be validated. It is worth noting that, since EH involves both catalytic and hydrolytic reactions, and NAC4ED is designed to parameterize the complex catalytic process, the design strategy of NAC4ED focuses on the nucleophilic attack reaction in the rate‐limiting step, while the effect of the hydrolytic reaction is not considered. This approach may impact the accuracy of the design. For example, in the case of the W117A mutation, despite a high share of active conformations, we believe that the alanine mutation may have destabilized the hydrogen bonding in the active pocket. This destabilization could lead to a significant reduction in the efficiency of the reaction involving the intermediate and activated water molecules. Additionally, the computational efficiency of the NAC4ED platform was evaluated. Under our conditions, utilizing a combination of one GPU (NVIDIA GeForce RTX 3090) and one CPU (Xeon Gold 6138, 20 cores), we found that obtaining the population of active conformations for one variant required only 13 min. In comparison, manually completing the modeling of one enzyme variant took nearly 50 min owing to the laborious process of structural manipulation and file preparation, in addition to the computational runtime (Figure 4C). On the other hand, experimental characterization of one enzyme variant, involving steps such as gene mutation, sequencing validation, host transformation, protein expression, protein purification, and final enzyme activity assays, required approximately 7 days to complete^44^, ^45^, consuming 764 times more time than the NAC4ED platform (Figure 4D). We automated the evaluation of active conformation populations of 1843 variants using two CPUs (40 cores) and two GPUs within 192 h, which was attributable to the use of NAC rather than QM/MM calculations during simulation. Meanwhile, experimental characterization methods are limited in parallelization. For the 1843 variants, experimental characterization would require 12,901 days, which is 1613 times longer than the NAC4ED platform. It is worth noting that the computational efficiency for evaluating 1843 variants is limited by resource constraints. If deployed on a larger computing cluster (40 GPUs, 1600 CPU cores), the NAC4ED platform's computational time could be reduced to 10 h. Therefore, under computing resource conditions, the NAC4ED platform can increase the efficiency of mutation screening by tens of thousands of times. Furthermore, with ample computational resources, the larger the variant space to be evaluated, the higher the screening efficiency of NAC4ED, potentially reaching hundreds of thousands or even millions of times, significantly reducing the cost of experimental characterization and facilitating the discovery of amino acid mutations that enhance catalytic activity. It is worth noting, however, that we do not intend to overstate the impact of the current results, as rankings based on NAC may not fully coincide with experimental validation results, and the expression and solubility of mutant variants have not been considered. Additionally, it is worth mentioning that during MD sampling, the system may be prone to becoming stuck in local optimal structures, or the sample size of the accessible space may not be satisfactory. In practice, we recommend that users conduct benchmark tests tailored to specific systems to optimize simulation settings.

### Generic validation of the NAC4ED platform

This study further validates the universality of the NAC4ED platform. Amine transaminases (ATAs) are powerful biocatalysts for the stereoselective synthesis of chiral amines^46^. Based on the structure and catalytic mechanism of the transaminase 3FCR, Ao et al.^47^ collected data on the catalytic performance of key amino acid mutants. We utilized a data set from the literature, which included 40 3FCR mutants for the catalytic activity of S‐phenylethylamine. Using NAC4ED software, single and combination mutations were evaluated to determine the transferability and accuracy of NAC4ED. By determining the docking conformations of the 3FXR enzyme and substrate molecules and conducting molecular dynamics simulations, we performed computational screening for 40 combined mutations. The populations of active conformations for each mutation are provided in Table S1. In comparison to the WT, there were 15 mutations experimentally determined to have higher activity, whereas 25 mutations exhibited lower activity. In contrast, NAC4ED predicted 13 mutations with high activity and 24 mutations with low activity, with a prediction accuracy of 92.5%. It is noteworthy that the overall trend of the computational results is consistent with the experimental findings. Through this computational experiment, the broad applicability of the NAC4ED platform was confirmed. Furthermore, in most cases, the computational complexity of high‐throughput screening of enzyme mutations is significantly reduced compared to expensive QM/MM calculations. Compared to machine learning methods, which are constrained by the availability of high‐quality training data and specific models, NAC4ED offers a broader generalization based on the physical principles of enzyme‐catalyzed reactions. It allows for amino acid design across any enzyme reaction. Additionally, NAC4ED can serve as a feature generator for machine learning. The key catalytic parameters and results it generates can be incorporated into the training process as machine‐learning features, thereby integrating physics‐based computational methods with machine‐learning techniques. These results demonstrate the strong potential of the NAC4ED platform for rapidly verifying the fitness landscape of enzyme mutations.

In summary, we develope a high‐performance, high‐throughput enzyme mutagenesis computational design platform called NAC4ED based on the “NAC” strategy. It integrates modeling, docking, and simulation software to automate the enzyme mutagenesis design cycle. Compared to QM/MM design, this work reduces time costs while maintaining accuracy by combining “NAC”, docking, and MD simulation, enabling high‐throughput screening of enzymes. Currently, NAC4ED has been uploaded to http://lujialab.org.cn/software/. NAC4ED facilitates the rapid screening and identification of beneficial enzyme variants, accelerating the development of novel biocatalysts for catalyzing nonnative substrates. Additionally, NAC4ED contributes to the generation of computational data for enzyme databases, providing guidance for future statistical modeling and machine‐learning endeavors in the field of enzymology.

## MATERIALS AND METHODS

### Software operation

A mutant module was first developed to generate computational models of enzyme variants in a random or user‐defined manner. The mutant score function creates a list of mutations in a user‐defined manner. The desired mutations are specified by providing a list of “X#Y” tokens, where X refers to the residue before the mutation, # refers to the residue index, and Y refers to the mutated residue. The module integrates pymol with AMBER scripts. Mutant enzyme conformations are sampled by molecular dynamics methods to optimize variant structures. For the docking module, we used the glide program to dock the substrate to the enzyme and determine the 20 × 20 × 20 ligand box based on the key amino acid residues provided. The LigPrep program, was also used for optimization of substrate molecules. Molecular dynamics module, the tLEaP program, was used to process the docked system, minimized using the steepest descent method with conjugate gradient method, and performed 500 ps MD of the NPT system without any constraints. The evaluation analysis module performs analysis of key parameters, such as distances and angles, using the CPPTRAJ program.

### Plasmid construction

The TrEH constructed in PET‐28b was chosen as the template for constructing mutants using PCR. Primer design depended on the specific amino acid chosen. Typically, a 25 µl reaction mixture contained 11 µl water, 12.5 µl PrimeSTAR Max Premix (2×), 0.5 µl template DNA (50–100 ng), and 10 µM primer mix (1 µl). PCR conditions were as follows: 98°C for 5 min, followed by 35 cycles (98°C for 10 s, 58°C for 15 s, and 72°C for 60 s), and a final extension at 72°C for 10 min. PCR products were analyzed by agarose gel electrophoresis. *Dpn*I (0.2 µl) was added to a 10 µl PCR reaction mixture and digested at 37°C for 1 h. The digested PCR products were transformed into *Escherichia coli* cloning hosts.

### Protein expression

*E. coli* BL21 (DE3) cells carrying recombinant plasmids were cultured in 10 ml LB medium containing kanamycin (50 µg/ml) overnight at 37°C. The overnight culture was inoculated into 400 ml LB medium supplemented with kanamycin and grown at 37°C. When the OD_600_ reached 0.6, IPTG was added to a final concentration of 0.2 mM to induce protein expression, followed by further growth at 20°C for 12 h. After centrifugation at 8000*g* for 10 min at 4°C, the bacterial pellets were washed once with phosphate‐buffered saline (50 mM, pH 7.4), resuspended in Tris‐HCl buffer (50 mM, pH 7.4), and then sonicated. The crude enzyme solution was purified using a Ni gravity column.

### Styrene oxide hydrolysis activity assay

10 μl of 100 mM styrene oxide was added to 90 μl of enzyme solution and mixed thoroughly. Then, 100 μl of 100 mM Styrene oxide and 100 μl of the recombinant WT TrEH or mutant mixture were incubated at 30°C for 20 min. After adding 100 μl of 4‐(4‐nitrobenzyl) pyridine, the mixture was stirred at 80°C for 10 min and cooled in cold water. Then, 100 μl of the mixture was combined with 100 μl of acetone and triethylamine, and the absorbance was measured at 565 nm.

## AUTHOR CONTRIBUTIONS

**Chuanxi Zhang**: Data curation (lead); formal analysis (lead); investigation (lead); methodology (lead); software (lead); writing—original draft (lead); writing—review and editing (equal). **Yinghui Feng**: Data curation (equal); validation (equal); writing—review and editing (equal). **Yiting Zhu**: Validation (equal). **Lei Gong**: Visualization (lead); writing—review and editing (equal). **Hao Wei**: Supervision (equal); writing—review and editing (equal). **Lujia Zhang**: Funding acquisition (lead); investigation (supporting); project administration (lead); resources (equal); supervision (lead); writing—review and editing (lead).

## ETHICS STATEMENT

This article does not contain any studies with human participants or animals performed by any of the authors.

## CONFLICT OF INTERESTS

The authors declare no conflict of interests.

## Supporting information

Supporting information.

## Data Availability

All data relevant to this article have been provided in the Supporting Information; any additional information required for reanalyzing the data reported in this article is available from the lead contact upon request. All materials generated in this study are available from the lead contact. Further information and requests for resources should be directed to and will be fulfilled by the lead contact, Lujia Zhang (Ljzhang@chem.ecnu.edu.cn).
