# Correction to “NAC4ED: A high‐throughput computational platform for the rational design of enzyme activity and substrate selectivity”

**DOI:** 10.1002/mlf2.70000

**Published:** 2025-03-27

Zhang C, Feng Y, Zhu Y, Gong L, Wei H, Zhang L. NAC4ED: a high‐throughput computational platform for the rational design of enzyme activity and substrate selectivity. mLife. 2024;3:505–514. https://doi.org/10.1002/mlf2.12154

The caption of Figure 3 is missing. The figure caption should be: Processes and modules of NAC4ED.

We apologize for this error.

