# Supplementary material for: NAC4ED: A high‐throughput computational platform for the rational design of enzyme activity and substrate selectivity
Source: mLife. 2024 Dec 25;3(4):505–14. doi: 10.1002/mlf2.12154 (PMC11685835; doi:10.1002/mlf2.12154)
Supplement: Supplementary file 1 — Supporting information. [file MLF2-3-505-s001.docx]

**NAC4ED: A high-throughput computational platform for the rational design of enzyme activity and substrate selectivity**

**Chuanxi Zhang^a^, Yinghui Feng^b^, Yiting Zhu^d^, Lei Gong^e^, Hao Wei^a^, Lujia Zhang^b,c,*^**

*^a^ Department of Micro/Nano Electronics, School of Electronic Information and Electrical Engineering, Shanghai Jiao Tong University, Shanghai, 200240, China*

*^b^ Shanghai Engineering Research Center of Molecular Therapeutics & New Drug Development, School of Chemistry and Molecular Engineering, East China Normal University, Shanghai 200062, China*

*^c^ NYU-ECNU Center for Computational Chemistry at NYU Shanghai, Shanghai 200062, China*

*^d^ School of Biotechnology, East China University of Science and Technology, Shanghai 200237, China*

*^e^ School of Biotechnology, Tianjin University of Science and Technology, Tianjin 300457, China*

*Corresponding authors: Shanghai Engineering Research Center of Molecular Therapeutics & New Drug Development, School of Chemistry and Molecular Engineering, East China Normal University, Shanghai 200062, China

**Fig. S1.** SO hydrolysis standard curve determination

**Fig.S2.** Modeling quality assessment

**Table S1.** Calculation of the conformational bougie number of transaminase activity

**Table S2.** Evaluation of the active conformational population of 1843 variants


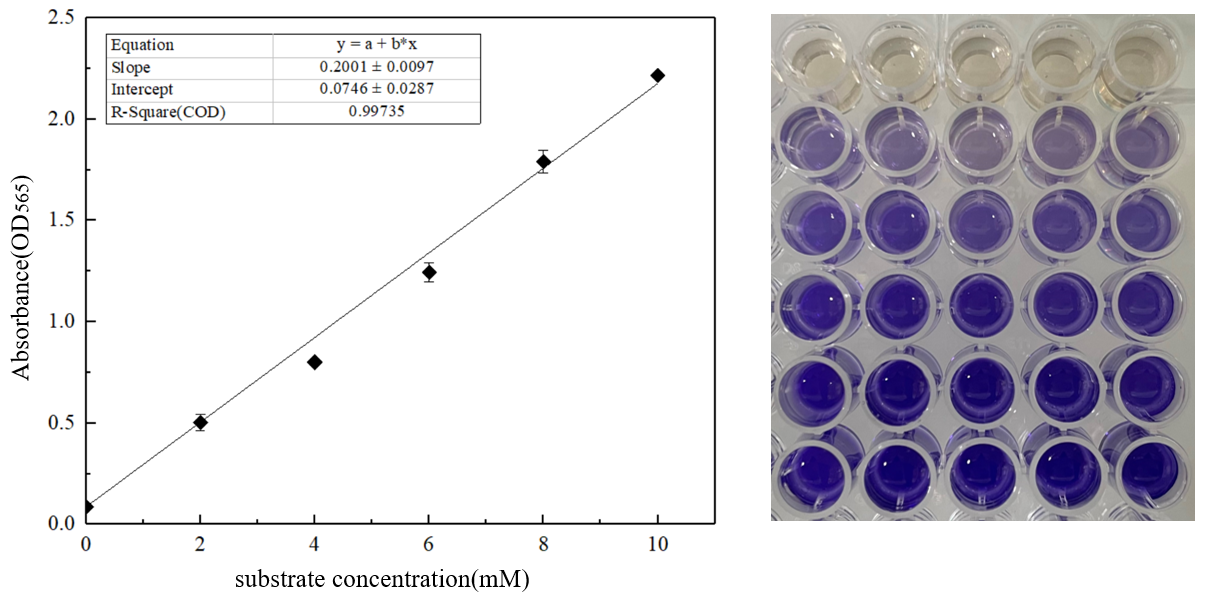


**Fig.S1.** SO hydrolysis standard curve determination


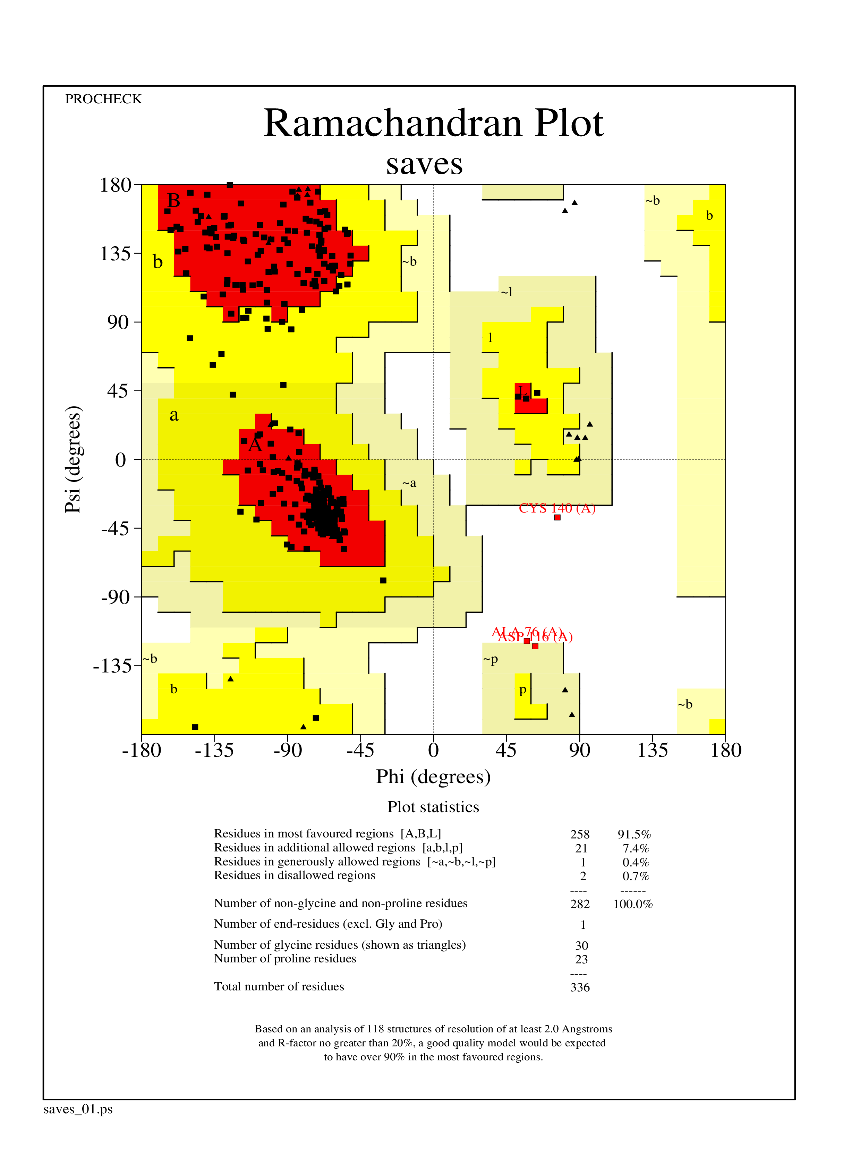


**Fig.S2.** Modeling quality assessment

**Table S1.** Calculation of the conformational bougie number of transaminase activity

| Variants | Specific activity (U mg^-1^) | Activity Conformation ratio(%) |
| --- | --- | --- |
| Y87F/T231A/Y59W | 19.2581 | 95.5 |
| Y87F/T231A/Y59W/Y152F | 7.2601 | 97.1 |
| Y87F/S19W/T231A/Y59W | 5.6152 | 83.2 |
| Y87F/S19V/T231A/Y59W | 5.4472 | 85 |
| Y87F/T231A/Y59W/Y152F/L58V | 3.6568 | 74.4 |
| Y87F/T231L/Y59W/Y152F | 2.1375 | 38.8 |
| Y87L/S19W/T231A/Y59W | 1.9948 | 43.2 |
| Y87F/T231A/Y59W/Y152F/L58A | 1.4532 | 10.8 |
| Y87L/T231A/Y59W | 0.7622 | 52.5 |
| Y87F/S19V/T231L | 0.7033 | 14.7 |
| Y87F/S19F/T231L/Y59W/Y152F | 0.6932 | 98.5 |
| Y87L/S19W/T231L/Y59W | 0.6787 | 74.5 |
| Y87L/S19V/T231L/Y59W | 0.6754 | 66 |
| Y87L/S19W/T231L | 0.3502 | 72.4 |
| Y87L/S19W | 0.2821 | 4.1 |
| WT | 0.1863 | 37 |
| S19W/T231L | 0.1859 | 38.2 |
| Y87L/S19W/T231S | 0.1732 | 32.3 |
| Y87F/T231L/Y59W/Y152F | 0.1711 | 25.2 |
| T231L | 0.1613 | 9 |
| Y87L/S19W/Y152F | 0.1559 | 7.7 |
| L58V | 0.1396 | 24 |
| Y87F | 0.1163 | 25 |
| Y152L/S155G | 0.1019 | 22.3 |
| Y152F | 0.0999 | 21 |
| Y87L/S19W/T231A | 0.0992 | 29 |
| Y87L/S19V/T231M | 0.0524 | 18 |
| Y87L/S19V/T231L | 0.0503 | 5 |
| Y87L/T231A | 0.0451 | 16.5 |
| Y87F/Y152F | 0.0326 | 0.2 |
| Y87L/T231G | 0.0324 | 14 |
| Y87L/S19A | 0.0134 | 12 |
| Y87L/Y152F | 0.0099 | 8 |
| Y87L | 0.007 | 3 |
| Y87L/S19V | 0.0068 | 15.1 |
| Y87L/S19W/T231P | 0.0057 | 0.1 |
| Y87L/S19W/T231H | 0.0052 | 9.71 |
| Y87L/S19W/T231Q | 0.0044 | 0.4 |
| Y87L/Y152F/S155A | 0.0035 | 2 |
| Y87F/Y152L/S155G | 0.0034 | 0.2 |
| Y87L/S19V/T231Q | 0.0023 | 0.6 |

Table S2. Evaluation of the active conformational population of 1843 variants

| Mutation | Ratio（%） | Mutation | Ratio（%） | Mutation | Ratio（%） | Mutation | Ratio（%） |
| --- | --- | --- | --- | --- | --- | --- | --- |
| G119I | 24.3 | M193W | 4.6 | W314N | 4.3 | S208R | 3.9 |
| L89Y | 20.9 | S206L | 4.6 | M49L | 4.2 | P222W | 3.9 |
| L89F | 16 | L216W | 4.6 | L89H | 4.2 | R253Y | 3.9 |
| H144E | 12 | A288C | 4.6 | W117K | 4.2 | F280A | 3.9 |
| F165S | 9.6 | L169M | 4.5 | L153K | 4.2 | A288L | 3.9 |
| W117A | 8.7 | L186I | 4.5 | L153I | 4.2 | L289C | 3.9 |
| W117H | 7.6 | T207F | 4.5 | H144Q | 4.2 | M293T | 3.9 |
| H144L | 6.6 | S208W | 4.5 | L186W | 4.2 | L316Y | 3.9 |
| H144W | 6.1 | L249P | 4.5 | R197P | 4.2 | H44I | 3.8 |
| P47K | 5.6 | N287L | 4.5 | I219K | 4.2 | G114K | 3.8 |
| Y75R | 5.6 | L289W | 4.5 | P222A | 4.2 | H144F | 3.8 |
| L224W | 5.6 | D48F | 4.4 | L245M | 4.2 | H144S | 3.8 |
| Y237W | 5.6 | G119K | 4.4 | R255L | 4.2 | R188K | 3.8 |
| Y75K | 5.5 | C140H | 4.4 | A282W | 4.2 | V211L | 3.8 |
| L316N | 5.4 | L153R | 4.4 | A311I | 4.2 | L216K | 3.8 |
| F190W | 5.2 | F165A | 4.4 | W117I | 4.1 | P223N | 3.8 |
| F194L | 5.2 | F165Y | 4.4 | Y167A | 4.1 | L224P | 3.8 |
| G210W | 5.2 | L186F | 4.4 | Y167M | 4.1 | W251P | 3.8 |
| R197C | 5 | R188F | 4.4 | F194K | 4.1 | R253F | 3.8 |
| L89P | 4.9 | R188M | 4.4 | F205K | 4.1 | K285W | 3.8 |
| L153H | 4.9 | F189W | 4.4 | S206E | 4.1 | D286F | 3.8 |
| V176L | 4.9 | G195W | 4.4 | Y233I | 4.1 | N287W | 3.8 |
| F189L | 4.9 | A203H | 4.4 | N250L | 4.1 | A288M | 3.8 |
| Y252L | 4.9 | D209K | 4.4 | D48L | 4 | P290A | 3.8 |
| M162K | 4.8 | L230P | 4.4 | K166R | 4 | P290C | 3.8 |
| Y167L | 4.8 | G247W | 4.4 | Y167P | 4 | A311F | 3.8 |
| G210A | 4.8 | W251M | 4.4 | V176A | 4 | T312D | 3.8 |
| P248A | 4.8 | A283L | 4.4 | G210M | 4 | W314A | 3.8 |
| H313S | 4.8 | S284L | 4.4 | A283C | 4 | H44R | 3.7 |
| A315C | 4.8 | M293P | 4.4 | K285L | 4 | H44F | 3.7 |
| W46A | 4.7 | L186K | 4.3 | L289P | 4 | H44L | 3.7 |
| M49F | 4.7 | H44K | 4.3 | T312H | 4 | L163F | 3.7 |
| R191F | 4.7 | M49H | 4.3 | R188I | 3.9 | L163P | 3.7 |
| D209R | 4.7 | A120L | 4.3 | G114H | 3.9 | N164M | 3.7 |
| P223C | 4.7 | Y167W | 4.3 | G118K | 3.9 | F165M | 3.7 |
| L225K | 4.7 | L186H | 4.3 | A120W | 3.9 | F165D | 3.7 |
| D48I | 4.6 | R188H | 4.3 | Q170K | 3.9 | K166I | 3.7 |
| L171F | 4.6 | F194W | 4.3 | V176M | 3.9 | K166F | 3.7 |
| H144P | 4.6 | R197N | 4.3 | R188P | 3.9 | Q168L | 3.7 |
| R191W | 4.6 | A203K | 4.3 | F189A | 3.9 | V176P | 3.7 |
| R191A | 4.6 | R255M | 4.3 | R191C | 3.9 | L186A | 3.7 |
| A192L | 4.6 | H313E | 4.3 | T207A | 3.9 | R188C | 3.7 |
| Mutation | Ratio（%） | Mutation | Ratio（%） | Mutation | Ratio（%） | Mutation | Ratio（%） |
| F190Q | 3.7 | T207H | 3.5 | R54A | 3.3 | P290N | 3.2 |
| L224K | 3.7 | T207M | 3.5 | L89I | 3.3 | P291L | 3.2 |
| P248M | 3.7 | G210R | 3.5 | L89M | 3.3 | M293R | 3.2 |
| N250W | 3.7 | I219H | 3.5 | H115I | 3.3 | T312G | 3.2 |
| N250A | 3.7 | P222I | 3.5 | A120M | 3.3 | H313K | 3.2 |
| K285A | 3.7 | P223V | 3.5 | V121R | 3.3 | A315G | 3.2 |
| W314V | 3.7 | L224C | 3.5 | T141H | 3.3 | T317W | 3.2 |
| A315V | 3.7 | L230F | 3.5 | L153F | 3.3 | W117R | 3.1 |
| L316P | 3.7 | L230C | 3.5 | R188N | 3.3 | A288H | 3.1 |
| L316G | 3.7 | W251C | 3.5 | A203P | 3.3 | V43R | 3.1 |
| G119F | 3.6 | R253M | 3.5 | V211W | 3.3 | W46M | 3.1 |
| V121H | 3.6 | R253P | 3.5 | L225I | 3.3 | D48M | 3.1 |
| S138K | 3.6 | R255P | 3.5 | L225F | 3.3 | L89R | 3.1 |
| V139F | 3.6 | A282K | 3.5 | L245F | 3.3 | N164A | 3.1 |
| N164C | 3.6 | A283N | 3.5 | L245C | 3.3 | Y167C | 3.1 |
| K172A | 3.6 | L289I | 3.5 | L249I | 3.3 | Q168C | 3.1 |
| V176F | 3.6 | M293C | 3.5 | L249N | 3.3 | L171A | 3.1 |
| V176C | 3.6 | A315F | 3.5 | R255W | 3.3 | K172P | 3.1 |
| M193R | 3.6 | D48A | 3.4 | R255C | 3.3 | F190M | 3.1 |
| G195I | 3.6 | Q168P | 3.4 | A283H | 3.3 | R191I | 3.1 |
| G204W | 3.6 | V176N | 3.4 | L289N | 3.3 | F194A | 3.1 |
| H212M | 3.6 | T207P | 3.4 | P290K | 3.3 | G204A | 3.1 |
| P223M | 3.6 | S208E | 3.4 | T312Q | 3.3 | S206A | 3.1 |
| Y233F | 3.6 | G210P | 3.4 | H313V | 3.3 | V211R | 3.1 |
| G247R | 3.6 | P222R | 3.4 | H313G | 3.3 | V211F | 3.1 |
| A283I | 3.6 | P223G | 3.4 | Y75I | 3.2 | L225W | 3.1 |
| M293F | 3.6 | G247A | 3.4 | A120P | 3.2 | L225A | 3.1 |
| L316A | 3.6 | P248R | 3.4 | S138I | 3.2 | Y237N | 3.1 |
| A120R | 3.5 | L249C | 3.4 | T141I | 3.2 | Y237V | 3.1 |
| L89N | 3.5 | Y252H | 3.4 | M162I | 3.2 | L249V | 3.1 |
| V139I | 3.5 | R255F | 3.4 | N164W | 3.2 | N250R | 3.1 |
| P142F | 3.5 | A283P | 3.4 | L171I | 3.2 | N250F | 3.1 |
| F165H | 3.5 | S284K | 3.4 | R197G | 3.2 | W251N | 3.1 |
| L169C | 3.5 | S284W | 3.4 | T207K | 3.2 | F280I | 3.1 |
| K172M | 3.5 | A288N | 3.4 | Y233L | 3.2 | A282L | 3.1 |
| F189K | 3.5 | A311H | 3.4 | Y237C | 3.2 | S284M | 3.1 |
| A192R | 3.5 | H313R | 3.4 | Y252W | 3.2 | N287R | 3.1 |
| M193A | 3.5 | A315W | 3.4 | R255N | 3.2 | N287A | 3.1 |
| R197I | 3.5 | T317I | 3.4 | S284A | 3.2 | A311Y | 3.1 |
| R197V | 3.5 | A120K | 3.3 | D286R | 3.2 | H313I | 3.1 |
| A203M | 3.5 | D48K | 3.3 | D286I | 3.2 | V43K | 3 |
| S206W | 3.5 | G52R | 3.3 | L289A | 3.2 | G114I | 3 |
| Mutation | Ratio（%） | Mutation | Ratio（%） | Mutation | Ratio（%） | Mutation | Ratio（%） |
| L153W | 3 | N287K | 2.9 | F280D | 2.8 | A283K | 2.7 |
| L163C | 3 | A288W | 2.9 | A288P | 2.8 | A288K | 2.7 |
| L169N | 3 | P290I | 2.9 | L289V | 2.8 | L289G | 2.7 |
| A192W | 3 | P291F | 2.9 | A311K | 2.8 | P291A | 2.7 |
| S206M | 3 | P291W | 2.9 | W314M | 2.8 | M293I | 2.7 |
| D209I | 3 | M293W | 2.9 | A315M | 2.8 | M293A | 2.7 |
| R253C | 3 | T312V | 2.9 | A315S | 2.8 | A311T | 2.7 |
| R255V | 3 | T312Y | 2.9 | T317F | 2.8 | L316V | 2.7 |
| A311L | 3 | T312E | 2.9 | T317E | 2.8 | Q318D | 2.7 |
| T312R | 3 | W314G | 2.9 | Q318I | 2.8 | V43I | 2.6 |
| T317M | 3 | L316W | 2.9 | Q318L | 2.8 | W46F | 2.6 |
| Q318G | 3 | T317R | 2.9 | Q318M | 2.8 | D48R | 2.6 |
| L216I | 2.9 | T317P | 2.9 | Q318P | 2.8 | D48P | 2.6 |
| M49W | 2.9 | Q318W | 2.9 | G118H | 2.7 | M72W | 2.6 |
| G119L | 2.9 | A120H | 2.8 | G118I | 2.7 | W117F | 2.6 |
| T141W | 2.9 | G52K | 2.8 | C140I | 2.7 | G119H | 2.6 |
| L153A | 2.9 | M72F | 2.8 | T141A | 2.7 | V121W | 2.6 |
| N164G | 2.9 | H115R | 2.8 | Y167F | 2.7 | L163K | 2.6 |
| Q168F | 2.9 | V139H | 2.8 | L171K | 2.7 | Q168A | 2.6 |
| Q170R | 2.9 | P142K | 2.8 | K172L | 2.7 | K172R | 2.6 |
| F189M | 2.9 | P142L | 2.8 | H144Y | 2.7 | V176I | 2.6 |
| R197S | 2.9 | V176W | 2.8 | L186M | 2.7 | H144K | 2.6 |
| A203C | 2.9 | F189R | 2.8 | F189P | 2.7 | H144C | 2.6 |
| H212I | 2.9 | F190H | 2.8 | R191K | 2.7 | F190P | 2.6 |
| L216R | 2.9 | F190I | 2.8 | M193P | 2.7 | A192M | 2.6 |
| L216M | 2.9 | A192H | 2.8 | F194H | 2.7 | M193H | 2.6 |
| P222K | 2.9 | G195K | 2.8 | R197A | 2.7 | M193I | 2.6 |
| P223L | 2.9 | G195P | 2.8 | G204L | 2.7 | M193C | 2.6 |
| L225H | 2.9 | T207C | 2.8 | F205I | 2.7 | F194I | 2.6 |
| L225M | 2.9 | S208F | 2.8 | S206K | 2.7 | G195H | 2.6 |
| Y237A | 2.9 | G210H | 2.8 | T207N | 2.7 | R197Q | 2.6 |
| P248W | 2.9 | G210L | 2.8 | S208L | 2.7 | F205L | 2.6 |
| W251I | 2.9 | V211K | 2.8 | S208M | 2.7 | S208P | 2.6 |
| W251L | 2.9 | L224R | 2.8 | D209H | 2.7 | V211A | 2.6 |
| Y252F | 2.9 | L225P | 2.8 | L230N | 2.7 | H212W | 2.6 |
| R253D | 2.9 | L230I | 2.8 | Y233R | 2.7 | L216P | 2.6 |
| R253T | 2.9 | G247M | 2.8 | L245N | 2.7 | P222F | 2.6 |
| S284F | 2.9 | P248C | 2.8 | L245V | 2.7 | L225R | 2.6 |
| K285I | 2.9 | L249H | 2.8 | G247H | 2.7 | L230M | 2.6 |
| K285M | 2.9 | N250I | 2.8 | R255I | 2.7 | Y233K | 2.6 |
| D286H | 2.9 | W251R | 2.8 | R255G | 2.7 | Y237P | 2.6 |
| D286L | 2.9 | Y252A | 2.8 | F280W | 2.7 | G247K | 2.6 |
| Mutation | Ratio（%） | Mutation | Ratio（%） | Mutation | Ratio（%） | Mutation | Ratio（%） |
| Y252R | 2.6 | M293H | 2.5 | T317C | 2.4 | R54F | 2.2 |
| R253A | 2.6 | M293N | 2.5 | Q318C | 2.4 | L89W | 2.2 |
| F280R | 2.6 | F190R | 2.4 | R54I | 2.3 | H115F | 2.2 |
| S284P | 2.6 | V43H | 2.4 | M72L | 2.3 | V121K | 2.2 |
| K285P | 2.6 | R54M | 2.4 | G114L | 2.3 | S138R | 2.2 |
| M293E | 2.6 | H115K | 2.4 | W117L | 2.3 | T141F | 2.2 |
| A311W | 2.6 | G119W | 2.4 | M162F | 2.3 | N164L | 2.2 |
| T312K | 2.6 | S138H | 2.4 | L163H | 2.3 | F165R | 2.2 |
| H313N | 2.6 | T141R | 2.4 | K166L | 2.3 | F165W | 2.2 |
| A315I | 2.6 | Q170H | 2.4 | L169W | 2.3 | L169P | 2.2 |
| A315L | 2.6 | L171M | 2.4 | L169V | 2.3 | L171P | 2.2 |
| L316M | 2.6 | F189I | 2.4 | Q170L | 2.3 | K172C | 2.2 |
| T317L | 2.6 | F190K | 2.4 | R188A | 2.3 | L186R | 2.2 |
| N287T | 2.5 | F190S | 2.4 | F194T | 2.3 | A192F | 2.2 |
| W46L | 2.5 | M193N | 2.4 | G195C | 2.3 | A203R | 2.2 |
| W46P | 2.5 | F194R | 2.4 | G196S | 2.3 | F205A | 2.2 |
| D48C | 2.5 | G195F | 2.4 | G204I | 2.3 | S206P | 2.2 |
| G114F | 2.5 | T207I | 2.4 | T207R | 2.3 | S208C | 2.2 |
| N164H | 2.5 | S208A | 2.4 | T207W | 2.3 | Y233M | 2.2 |
| Y167H | 2.5 | G210F | 2.4 | D209F | 2.3 | G247F | 2.2 |
| Q168W | 2.5 | I219F | 2.4 | H212L | 2.3 | G247P | 2.2 |
| Q170F | 2.5 | I219L | 2.4 | L225N | 2.3 | G247C | 2.2 |
| K172W | 2.5 | I219W | 2.4 | Y233A | 2.3 | R253I | 2.2 |
| H144N | 2.5 | P223A | 2.4 | P248F | 2.3 | R255Q | 2.2 |
| A192P | 2.5 | P223S | 2.4 | Y252M | 2.3 | F280K | 2.2 |
| R197M | 2.5 | L230R | 2.4 | R253V | 2.3 | F280M | 2.2 |
| A203N | 2.5 | Y233W | 2.4 | R255S | 2.3 | S284I | 2.2 |
| F205W | 2.5 | Y237H | 2.4 | A282I | 2.3 | D286A | 2.2 |
| S208K | 2.5 | L245I | 2.4 | A283F | 2.3 | A288F | 2.2 |
| V211H | 2.5 | L249K | 2.4 | A283W | 2.3 | P291R | 2.2 |
| L216A | 2.5 | L249W | 2.4 | A283V | 2.3 | T312I | 2.2 |
| P222M | 2.5 | L249G | 2.4 | N287F | 2.3 | H313Q | 2.2 |
| L225C | 2.5 | Y252I | 2.4 | N287P | 2.3 | L316S | 2.2 |
| Y237I | 2.5 | A282M | 2.4 | P290W | 2.3 | A120F | 2.1 |
| L245H | 2.5 | S284C | 2.4 | P290V | 2.3 | V43W | 2.1 |
| P248H | 2.5 | D286W | 2.4 | P290T | 2.3 | G45I | 2.1 |
| N250H | 2.5 | N287M | 2.4 | A315Q | 2.3 | G45F | 2.1 |
| R253N | 2.5 | L289S | 2.4 | L316C | 2.3 | M72A | 2.1 |
| S284R | 2.5 | P290R | 2.4 | A120I | 2.2 | M72P | 2.1 |
| K285F | 2.5 | A311D | 2.4 | F205R | 2.2 | G118R | 2.1 |
| N287H | 2.5 | H313D | 2.4 | V43F | 2.2 | V121F | 2.1 |
| L289K | 2.5 | L316I | 2.4 | V43L | 2.2 | V139K | 2.1 |
| Mutation | Ratio（%） | Mutation | Ratio（%） | Mutation | Ratio（%） | Mutation | Ratio（%） |
| C140R | 2.1 | P290G | 2 | T141L | 1.8 | A315Y | 1.8 |
| N164R | 2.1 | Q318Y | 2 | P142W | 1.8 | T317N | 1.8 |
| R188L | 2.1 | A203W | 1.9 | P142A | 1.8 | T317V | 1.8 |
| R191L | 2.1 | G52I | 1.9 | L153P | 1.8 | V43A | 1.7 |
| R191P | 2.1 | L89A | 1.9 | L163N | 1.8 | W46K | 1.7 |
| R191N | 2.1 | H115L | 1.9 | N164S | 1.8 | D48V | 1.7 |
| A192C | 2.1 | L153M | 1.9 | K166W | 1.8 | R54N | 1.7 |
| G196V | 2.1 | L163I | 1.9 | K166A | 1.8 | H115W | 1.7 |
| R197H | 2.1 | N164F | 1.9 | Y167G | 1.8 | W117M | 1.7 |
| A203I | 2.1 | Y167V | 1.9 | Y167S | 1.8 | V121M | 1.7 |
| F205M | 2.1 | Q168N | 1.9 | L169A | 1.8 | V121P | 1.7 |
| S206I | 2.1 | L169K | 1.9 | L171C | 1.8 | V139R | 1.7 |
| S206C | 2.1 | L169I | 1.9 | L171N | 1.8 | V139L | 1.7 |
| G210I | 2.1 | H144V | 1.9 | L186P | 1.8 | T141K | 1.7 |
| P223I | 2.1 | F189C | 1.9 | L186C | 1.8 | T141P | 1.7 |
| P223Q | 2.1 | G195R | 1.9 | A192N | 1.8 | P142R | 1.7 |
| Y237L | 2.1 | G195N | 1.9 | A192V | 1.8 | L153C | 1.7 |
| P248N | 2.1 | G204M | 1.9 | M193V | 1.8 | F165L | 1.7 |
| R255Y | 2.1 | F205P | 1.9 | F194P | 1.8 | F165P | 1.7 |
| F280P | 2.1 | S206F | 1.9 | A203V | 1.8 | F165E | 1.7 |
| A282R | 2.1 | D209L | 1.9 | G204P | 1.8 | F165T | 1.7 |
| A282P | 2.1 | G210N | 1.9 | F205C | 1.8 | K166M | 1.7 |
| A283R | 2.1 | H212P | 1.9 | S206N | 1.8 | K166P | 1.7 |
| P290E | 2.1 | H212C | 1.9 | D209W | 1.8 | Q168K | 1.7 |
| A311Q | 2.1 | L216C | 1.9 | H212N | 1.8 | Q168V | 1.7 |
| H313F | 2.1 | L224H | 1.9 | P222V | 1.8 | L169S | 1.7 |
| H313L | 2.1 | L224N | 1.9 | L230K | 1.8 | Q170W | 1.7 |
| W314C | 2.1 | N250K | 1.9 | Y237M | 1.8 | K172H | 1.7 |
| Q318H | 2.1 | N250M | 1.9 | Y237G | 1.8 | K172N | 1.7 |
| P47I | 2 | F280T | 1.9 | L245K | 1.8 | V176G | 1.7 |
| G118F | 2 | A283M | 1.9 | L245G | 1.8 | R188V | 1.7 |
| G118L | 2 | P290S | 1.9 | L245S | 1.8 | M193F | 1.7 |
| T141M | 2 | P291M | 1.9 | L249A | 1.8 | F194C | 1.7 |
| Y167N | 2 | M293K | 1.9 | W251F | 1.8 | G204C | 1.7 |
| L169G | 2 | L316D | 1.9 | F280L | 1.8 | G204N | 1.7 |
| R191V | 2 | T317K | 1.9 | A282N | 1.8 | G204V | 1.7 |
| F194M | 2 | T317A | 1.9 | D286M | 1.8 | F205N | 1.7 |
| G210C | 2 | Q318V | 1.9 | P290F | 1.8 | T207V | 1.7 |
| P222C | 2 | P47F | 1.8 | A311M | 1.8 | S208N | 1.7 |
| P222N | 2 | D48N | 1.8 | T312F | 1.8 | G210V | 1.7 |
| A282C | 2 | R54P | 1.8 | W314S | 1.8 | V211M | 1.7 |
| N287C | 2 | R54C | 1.8 | W314Q | 1.8 | P223F | 1.7 |
| Mutation | Ratio（%） | Mutation | Ratio（%） | Mutation | Ratio（%） | Mutation | Ratio（%） |
| P223Y | 1.7 | F190C | 1.6 | N164I | 1.5 | D286V | 1.5 |
| L225V | 1.7 | R191G | 1.6 | N164Q | 1.5 | A288G | 1.5 |
| L230A | 1.7 | G195V | 1.6 | F165V | 1.5 | P291C | 1.5 |
| Y233T | 1.7 | A203G | 1.6 | K166C | 1.5 | P291T | 1.5 |
| Y237S | 1.7 | G204S | 1.6 | K166N | 1.5 | M293G | 1.5 |
| G247N | 1.7 | S208V | 1.6 | K166V | 1.5 | A311P | 1.5 |
| G247V | 1.7 | D209A | 1.6 | Q168G | 1.5 | T312W | 1.5 |
| G247S | 1.7 | G210S | 1.6 | Q168S | 1.5 | W314R | 1.5 |
| P248K | 1.7 | V211P | 1.6 | Q170M | 1.5 | A315T | 1.5 |
| L249S | 1.7 | V211C | 1.6 | Q170P | 1.5 | Q318A | 1.5 |
| R253L | 1.7 | V211N | 1.6 | K172G | 1.5 | Q318T | 1.5 |
| R255D | 1.7 | H212F | 1.6 | V176S | 1.5 | H44W | 1.4 |
| F280C | 1.7 | H212V | 1.6 | L186N | 1.5 | G45L | 1.4 |
| S284N | 1.7 | I219A | 1.6 | R188G | 1.5 | R54K | 1.4 |
| D286P | 1.7 | P222G | 1.6 | F189V | 1.5 | H115M | 1.4 |
| N287I | 1.7 | L224V | 1.6 | A192G | 1.5 | W117P | 1.4 |
| N287V | 1.7 | G247Q | 1.6 | G195S | 1.5 | W117C | 1.4 |
| N287G | 1.7 | N250P | 1.6 | G195Q | 1.5 | G118T | 1.4 |
| A288V | 1.7 | F280N | 1.6 | R197L | 1.5 | G119M | 1.4 |
| P290L | 1.7 | F280E | 1.6 | A203S | 1.5 | A120C | 1.4 |
| P291E | 1.7 | A282T | 1.6 | A203Q | 1.5 | C140K | 1.4 |
| M293D | 1.7 | D286C | 1.6 | G204Q | 1.5 | P142C | 1.4 |
| A311S | 1.7 | N287S | 1.6 | S206V | 1.5 | L153V | 1.4 |
| A311E | 1.7 | N287Q | 1.6 | T207G | 1.5 | N164Y | 1.4 |
| T312L | 1.7 | A288I | 1.6 | S208G | 1.5 | F165G | 1.4 |
| H313T | 1.7 | L289Q | 1.6 | D209M | 1.5 | K166G | 1.4 |
| W314H | 1.7 | P291I | 1.6 | V211G | 1.5 | Q170C | 1.4 |
| T317G | 1.7 | M293V | 1.6 | H212G | 1.5 | Q170N | 1.4 |
| W46C | 1.6 | W314Y | 1.6 | L224M | 1.5 | L171V | 1.4 |
| W46N | 1.6 | A315D | 1.6 | L224G | 1.5 | V176Q | 1.4 |
| P47L | 1.6 | A315E | 1.6 | L224S | 1.5 | L186V | 1.4 |
| D48G | 1.6 | L316E | 1.6 | L225G | 1.5 | R188S | 1.4 |
| G52F | 1.6 | W46V | 1.5 | L249R | 1.5 | R191S | 1.4 |
| R54V | 1.6 | P47W | 1.5 | Y252K | 1.5 | A192S | 1.4 |
| V139W | 1.6 | D48S | 1.5 | Y252P | 1.5 | F194N | 1.4 |
| F165C | 1.6 | G52L | 1.5 | R253K | 1.5 | G196W | 1.4 |
| F165N | 1.6 | R54G | 1.5 | A282V | 1.5 | A203Y | 1.4 |
| Q170A | 1.6 | H115A | 1.5 | A282G | 1.5 | F205V | 1.4 |
| L171W | 1.6 | G119A | 1.5 | A283G | 1.5 | F205G | 1.4 |
| K172V | 1.6 | P142M | 1.5 | K285C | 1.5 | F205S | 1.4 |
| H144G | 1.6 | L153N | 1.5 | K285N | 1.5 | S206G | 1.4 |
| F189N | 1.6 | N164K | 1.5 | D286N | 1.5 | T207L | 1.4 |
| Mutation | Ratio（%） | Mutation | Ratio（%） | Mutation | Ratio（%） | Mutation | Ratio（%） |
| T207S | 1.4 | R191Q | 1.3 | V121C | 1.2 | P291S | 1.2 |
| L216N | 1.4 | G195Y | 1.3 | S138F | 1.2 | P291Q | 1.2 |
| P223D | 1.4 | G196E | 1.3 | S138L | 1.2 | H313C | 1.2 |
| L224Q | 1.4 | G204Y | 1.3 | K166Q | 1.2 | W314T | 1.2 |
| N250C | 1.4 | T207Q | 1.3 | K166Y | 1.2 | A315H | 1.2 |
| F280V | 1.4 | D209P | 1.3 | L171R | 1.2 | T317Q | 1.2 |
| A283S | 1.4 | V211S | 1.3 | K172Q | 1.2 | T317Y | 1.2 |
| A283Q | 1.4 | H212S | 1.3 | V176Y | 1.2 | H144M | 1.1 |
| L289F | 1.4 | H212Q | 1.3 | L186G | 1.2 | Y75H | 1.1 |
| L289Y | 1.4 | P222H | 1.3 | F190N | 1.2 | V43M | 1.1 |
| P290H | 1.4 | L225S | 1.3 | R191Y | 1.2 | G45W | 1.1 |
| T312A | 1.4 | Y233P | 1.3 | A192Q | 1.2 | P47A | 1.1 |
| H313W | 1.4 | Y233D | 1.3 | G195D | 1.2 | W117N | 1.1 |
| W314D | 1.4 | Y237R | 1.3 | G196A | 1.2 | G119P | 1.1 |
| L316H | 1.4 | L245Q | 1.3 | G196T | 1.2 | S138W | 1.1 |
| L316T | 1.4 | P248V | 1.3 | R197E | 1.2 | S138A | 1.1 |
| R188W | 1.3 | L249Q | 1.3 | A203D | 1.2 | V139A | 1.1 |
| H44A | 1.3 | R253G | 1.3 | A203E | 1.2 | V139M | 1.1 |
| W46G | 1.3 | F280G | 1.3 | F205Q | 1.2 | V139P | 1.1 |
| D48Q | 1.3 | A282H | 1.3 | T207Y | 1.2 | T141C | 1.1 |
| R54S | 1.3 | S284V | 1.3 | S208Q | 1.2 | M162A | 1.1 |
| R54Q | 1.3 | S284G | 1.3 | G210K | 1.2 | K166H | 1.1 |
| M72C | 1.3 | S284T | 1.3 | H212Y | 1.2 | K166D | 1.1 |
| Y75F | 1.3 | K285V | 1.3 | I219M | 1.2 | Y167R | 1.1 |
| G114W | 1.3 | D286G | 1.3 | P222S | 1.2 | Y167E | 1.1 |
| H115P | 1.3 | L289D | 1.3 | P222Q | 1.2 | Q170V | 1.1 |
| C140F | 1.3 | P291N | 1.3 | P222Y | 1.2 | Q170G | 1.1 |
| C140L | 1.3 | P291V | 1.3 | Y233E | 1.2 | L171S | 1.1 |
| L153G | 1.3 | W314E | 1.3 | Y237F | 1.2 | K172Y | 1.1 |
| M162L | 1.3 | A315P | 1.3 | P248G | 1.2 | V176D | 1.1 |
| M162W | 1.3 | L316R | 1.3 | P248S | 1.2 | L186S | 1.1 |
| N164D | 1.3 | T317S | 1.3 | P248Q | 1.2 | M193G | 1.1 |
| K166S | 1.3 | Q318E | 1.3 | W251V | 1.2 | F194V | 1.1 |
| Y167Q | 1.3 | W46S | 1.2 | Y252C | 1.2 | G195L | 1.1 |
| Y167D | 1.3 | W46Q | 1.2 | R253E | 1.2 | R197T | 1.1 |
| Q168M | 1.3 | G52W | 1.2 | A282S | 1.2 | G204D | 1.1 |
| L169Q | 1.3 | G114A | 1.2 | K285G | 1.2 | S208Y | 1.1 |
| L169Y | 1.3 | G114M | 1.2 | K285S | 1.2 | D209C | 1.1 |
| L171G | 1.3 | H115C | 1.2 | D286S | 1.2 | V211I | 1.1 |
| K172S | 1.3 | H115N | 1.2 | A288S | 1.2 | H212D | 1.1 |
| R188Q | 1.3 | G118W | 1.2 | L289E | 1.2 | I219P | 1.1 |
| F189G | 1.3 | A120N | 1.2 | P291G | 1.2 | L224Y | 1.1 |
| Mutation | Ratio（%） | Mutation | Ratio（%） | Mutation | Ratio（%） | Mutation | Ratio（%） |
| L230V | 1.1 | D48Y | 0.9 | H115V | 0.8 | G52H | 0.7 |
| L245Y | 1.1 | R54Y | 0.9 | P142H | 0.8 | L89C | 0.7 |
| G247L | 1.1 | M72N | 0.9 | M162P | 0.8 | V121N | 0.7 |
| W251G | 1.1 | G114P | 0.9 | M162C | 0.8 | S138P | 0.7 |
| R253S | 1.1 | W117V | 0.9 | F165K | 0.8 | L163W | 0.7 |
| A283Y | 1.1 | W117G | 0.9 | Y167I | 0.8 | L163G | 0.7 |
| S284Q | 1.1 | G119N | 0.9 | L171Q | 0.8 | F165I | 0.7 |
| K285Q | 1.1 | G119V | 0.9 | L171Y | 0.8 | Y167K | 0.7 |
| K285Y | 1.1 | V121I | 0.9 | V176H | 0.8 | Q168I | 0.7 |
| D286K | 1.1 | L153Q | 0.9 | V176R | 0.8 | Q168Y | 0.7 |
| D286Q | 1.1 | L163M | 0.9 | H144H | 0.8 | L169D | 0.7 |
| D286Y | 1.1 | L163V | 0.9 | L186Q | 0.8 | L171H | 0.7 |
| N287Y | 1.1 | Q168R | 0.9 | F189H | 0.8 | H144R | 0.7 |
| A288Q | 1.1 | Q170Y | 0.9 | F189T | 0.8 | R191H | 0.7 |
| A288Y | 1.1 | K172E | 0.9 | F190V | 0.8 | A192K | 0.7 |
| L289M | 1.1 | M193S | 0.9 | R191D | 0.8 | R197W | 0.7 |
| P290Q | 1.1 | G196D | 0.9 | A192Y | 0.8 | R197Y | 0.7 |
| A311C | 1.1 | F205Y | 0.9 | F194G | 0.8 | G204K | 0.7 |
| A311N | 1.1 | S206Q | 0.9 | G196L | 0.8 | S208I | 0.7 |
| H313Y | 1.1 | D209N | 0.9 | R197K | 0.8 | I219N | 0.7 |
| W314P | 1.1 | I219C | 0.9 | F205H | 0.8 | I219V | 0.7 |
| L316K | 1.1 | L224F | 0.9 | S206R | 0.8 | P223E | 0.7 |
| L316F | 1.1 | L230E | 0.9 | S206Y | 0.8 | Y233C | 0.7 |
| T317H | 1.1 | Y233Q | 0.9 | H212K | 0.8 | G247D | 0.7 |
| T317D | 1.1 | G247Y | 0.9 | H212E | 0.8 | W251K | 0.7 |
| G45A | 1 | L249T | 0.9 | P223R | 0.8 | F280S | 0.7 |
| G119C | 1 | N250E | 0.9 | L225T | 0.8 | A288R | 0.7 |
| S138M | 1 | Y252E | 0.9 | L230G | 0.8 | L289R | 0.7 |
| L153S | 1 | F280Q | 0.9 | Y237K | 0.8 | P291Y | 0.7 |
| Q170S | 1 | P290D | 0.9 | L245R | 0.8 | T312S | 0.7 |
| K172D | 1 | M293S | 0.9 | L245W | 0.8 | H313A | 0.7 |
| L216V | 1 | M293Q | 0.9 | P248Y | 0.8 | A315R | 0.7 |
| L249Y | 1 | T312M | 0.9 | N250D | 0.8 | Q318R | 0.7 |
| Y252N | 1 | H44P | 0.8 | W251H | 0.8 | Q318K | 0.7 |
| K285D | 1 | W46R | 0.8 | Y252V | 0.8 | Q318S | 0.7 |
| A311V | 1 | W46Y | 0.8 | R255K | 0.8 | H44C | 0.6 |
| Q318F | 1 | D48H | 0.8 | R255E | 0.8 | W46H | 0.6 |
| H44M | 0.9 | M49K | 0.8 | A282F | 0.8 | R54D | 0.6 |
| G45M | 0.9 | R54L | 0.8 | S284E | 0.8 | M72R | 0.6 |
| G45P | 0.9 | Y75L | 0.8 | K285R | 0.8 | Y75W | 0.6 |
| G45C | 0.9 | L89T | 0.8 | P291D | 0.8 | L89K | 0.6 |
| D48W | 0.9 | G114C | 0.8 | W314K | 0.8 | L89D | 0.6 |
| Mutation | Ratio（%） | Mutation | Ratio（%） | Mutation | Ratio（%） | Mutation | Ratio（%） |
| G114N | 0.6 | H44N | 0.5 | L249M | 0.5 | V211T | 0.4 |
| H115G | 0.6 | G45H | 0.5 | W251T | 0.5 | H212R | 0.4 |
| W117S | 0.6 | W46I | 0.5 | Y252G | 0.5 | I219G | 0.4 |
| V121A | 0.6 | W46D | 0.5 | R253W | 0.5 | I219E | 0.4 |
| C140W | 0.6 | P47M | 0.5 | R255A | 0.5 | P222E | 0.4 |
| T141N | 0.6 | P47C | 0.5 | F280Y | 0.5 | L225D | 0.4 |
| M162N | 0.6 | D48E | 0.5 | A283D | 0.5 | L230T | 0.4 |
| M162V | 0.6 | D48T | 0.5 | A283E | 0.5 | Y237T | 0.4 |
| L163A | 0.6 | L89E | 0.5 | S284H | 0.5 | L245P | 0.4 |
| L163S | 0.6 | G114R | 0.5 | S284D | 0.5 | L245E | 0.4 |
| N164P | 0.6 | G114V | 0.5 | A288D | 0.5 | P248I | 0.4 |
| Q170I | 0.6 | G118E | 0.5 | A311R | 0.5 | W251A | 0.4 |
| Q170D | 0.6 | A120T | 0.5 | H313M | 0.5 | Y252T | 0.4 |
| K172I | 0.6 | V121L | 0.5 | V43P | 0.4 | A282E | 0.4 |
| K172T | 0.6 | S138C | 0.5 | V43C | 0.4 | D286E | 0.4 |
| L186Y | 0.6 | S138N | 0.5 | G45N | 0.4 | L289T | 0.4 |
| F189E | 0.6 | C140A | 0.5 | G45V | 0.4 | P290M | 0.4 |
| F190L | 0.6 | M162R | 0.5 | P47N | 0.4 | P291H | 0.4 |
| R191E | 0.6 | M162G | 0.5 | G52A | 0.4 | A311G | 0.4 |
| G196F | 0.6 | L163Q | 0.5 | R54H | 0.4 | T312C | 0.4 |
| G196M | 0.6 | L163Y | 0.5 | M72V | 0.4 | T312N | 0.4 |
| G196Y | 0.6 | K166E | 0.5 | Y75A | 0.4 | W314F | 0.4 |
| S208D | 0.6 | L169H | 0.5 | Y75M | 0.4 | W314L | 0.4 |
| D209Y | 0.6 | Q170E | 0.5 | Y75P | 0.4 | A315N | 0.4 |
| P222T | 0.6 | L186D | 0.5 | L89V | 0.4 | L316Q | 0.4 |
| P223W | 0.6 | F189S | 0.5 | L89G | 0.4 | H144A | 0.3 |
| L230W | 0.6 | R191M | 0.5 | G118A | 0.4 | V43N | 0.3 |
| L230S | 0.6 | R197F | 0.5 | S138V | 0.4 | V43G | 0.3 |
| L230Q | 0.6 | A203F | 0.5 | V139C | 0.4 | V43S | 0.3 |
| Y233H | 0.6 | G204H | 0.5 | C140M | 0.4 | H44V | 0.3 |
| G247I | 0.6 | G204R | 0.5 | P142I | 0.4 | H44G | 0.3 |
| L249D | 0.6 | G204F | 0.5 | M162S | 0.4 | H44S | 0.3 |
| W251E | 0.6 | G204E | 0.5 | Q168H | 0.4 | P47V | 0.3 |
| R255T | 0.6 | H212A | 0.5 | L171D | 0.4 | P47G | 0.3 |
| A282Q | 0.6 | L216H | 0.5 | K172F | 0.4 | M49I | 0.3 |
| A282D | 0.6 | L216F | 0.5 | V176E | 0.4 | G52M | 0.3 |
| P290Y | 0.6 | I219R | 0.5 | R188Y | 0.4 | G52P | 0.3 |
| P291K | 0.6 | P222L | 0.5 | F189D | 0.4 | G52C | 0.3 |
| M293L | 0.6 | L224I | 0.5 | F190T | 0.4 | M72K | 0.3 |
| T312P | 0.6 | L224A | 0.5 | R191T | 0.4 | M72G | 0.3 |
| W314I | 0.6 | L230H | 0.5 | D209V | 0.4 | Y75C | 0.3 |
| M193L | 0.5 | L230Y | 0.5 | G210T | 0.4 | Y75N | 0.3 |
| Mutation | Ratio（%） | Mutation | Ratio（%） | Mutation | Ratio（%） | Mutation | Ratio（%） |
| Y75V | 0.3 | A315K | 0.3 | A120S | 0.2 | V211D | 0.2 |
| G118M | 0.3 | V43Q | 0.2 | V121G | 0.2 | H212T | 0.2 |
| G118P | 0.3 | H44Q | 0.2 | V121S | 0.2 | L216D | 0.2 |
| G119S | 0.3 | H44Y | 0.2 | S138Q | 0.2 | I219S | 0.2 |
| G119Q | 0.3 | H44T | 0.2 | C140T | 0.2 | I219D | 0.2 |
| S138G | 0.3 | G45S | 0.2 | L153E | 0.2 | I219T | 0.2 |
| V139N | 0.3 | G45Q | 0.2 | M162H | 0.2 | P223H | 0.2 |
| V139G | 0.3 | G45T | 0.2 | M162Y | 0.2 | P223K | 0.2 |
| P142N | 0.3 | P47H | 0.2 | N164V | 0.2 | P223T | 0.2 |
| L153Y | 0.3 | P47R | 0.2 | N164E | 0.2 | L225E | 0.2 |
| L153D | 0.3 | P47S | 0.2 | F165Q | 0.2 | Y233N | 0.2 |
| M162Q | 0.3 | P47Q | 0.2 | Y167T | 0.2 | Y233S | 0.2 |
| L169R | 0.3 | M49R | 0.2 | Q168D | 0.2 | L245A | 0.2 |
| L169F | 0.3 | M49A | 0.2 | Q168E | 0.2 | L245T | 0.2 |
| L171E | 0.3 | M49P | 0.2 | Q168T | 0.2 | G247E | 0.2 |
| V176K | 0.3 | M49C | 0.2 | L169E | 0.2 | P248L | 0.2 |
| A192D | 0.3 | G52N | 0.2 | Q170T | 0.2 | P248E | 0.2 |
| M193T | 0.3 | G52V | 0.2 | L171T | 0.2 | L249F | 0.2 |
| F194E | 0.3 | G52S | 0.2 | H144D | 0.2 | N250V | 0.2 |
| G195A | 0.3 | G52Q | 0.2 | R188T | 0.2 | N250Q | 0.2 |
| G196N | 0.3 | G52Y | 0.2 | F189Q | 0.2 | W251Q | 0.2 |
| G196Q | 0.3 | R54W | 0.2 | F190G | 0.2 | Y252Q | 0.2 |
| F205D | 0.3 | R54E | 0.2 | F190Y | 0.2 | Y252D | 0.2 |
| G210Q | 0.3 | R54T | 0.2 | A192E | 0.2 | R253H | 0.2 |
| G210E | 0.3 | M72H | 0.2 | M193K | 0.2 | K285H | 0.2 |
| V211E | 0.3 | M72I | 0.2 | M193Y | 0.2 | N287D | 0.2 |
| L216G | 0.3 | M72S | 0.2 | M193E | 0.2 | N287E | 0.2 |
| L216Y | 0.3 | M72Q | 0.2 | F194S | 0.2 | L289H | 0.2 |
| I219Y | 0.3 | M72Y | 0.2 | F194D | 0.2 | Q318N | 0.2 |
| L224D | 0.3 | Y75G | 0.2 | G195E | 0.2 | A192I | 0.1 |
| L225Q | 0.3 | Y75S | 0.2 | G196H | 0.2 | V43Y | 0.1 |
| L230D | 0.3 | L89S | 0.2 | G196I | 0.2 | V43D | 0.1 |
| Y237E | 0.3 | L89Q | 0.2 | G196P | 0.2 | H44D | 0.1 |
| P248D | 0.3 | G114S | 0.2 | R197D | 0.2 | H44E | 0.1 |
| N250Y | 0.3 | H115S | 0.2 | G204T | 0.2 | G45R | 0.1 |
| W251S | 0.3 | H115Q | 0.2 | F205E | 0.2 | G45K | 0.1 |
| W251Y | 0.3 | G118C | 0.2 | S206D | 0.2 | G45Y | 0.1 |
| Y252S | 0.3 | G118N | 0.2 | T207E | 0.2 | G45D | 0.1 |
| R255H | 0.3 | G118V | 0.2 | S208H | 0.2 | G45E | 0.1 |
| K285E | 0.3 | G119Y | 0.2 | S208T | 0.2 | W46E | 0.1 |
| A288T | 0.3 | A120V | 0.2 | D209G | 0.2 | W46T | 0.1 |
| H313P | 0.3 | A120G | 0.2 | G210Y | 0.2 | P47Y | 0.1 |
| Mutation | Ratio（%） | Mutation | Ratio（%） | Mutation | Ratio（%） | Mutation | Ratio（%） |
| P47D | 0.1 | G119E | 0.1 | H144T | 0.1 | V43T | 0 |
| P47E | 0.1 | G119T | 0.1 | L186E | 0.1 | S138T | 0 |
| P47T | 0.1 | A120Q | 0.1 | L186T | 0.1 | V139D | 0 |
| M49N | 0.1 | A120Y | 0.1 | R188D | 0.1 | V139E | 0 |
| M49V | 0.1 | A120D | 0.1 | R188E | 0.1 | V139T | 0 |
| M49G | 0.1 | A120E | 0.1 | F189Y | 0.1 | C140V | 0 |
| M49S | 0.1 | V121Q | 0.1 | F190A | 0.1 | C140G | 0 |
| M49Q | 0.1 | V121Y | 0.1 | F190D | 0.1 | C140S | 0 |
| M49Y | 0.1 | V121D | 0.1 | F190E | 0.1 | C140Q | 0 |
| M49D | 0.1 | V121E | 0.1 | A192T | 0.1 | C140Y | 0 |
| M49E | 0.1 | V121T | 0.1 | M193D | 0.1 | C140D | 0 |
| M49T | 0.1 | S138Y | 0.1 | F194Q | 0.1 | C140E | 0 |
| G52D | 0.1 | S138D | 0.1 | G195M | 0.1 | T141Q | 0 |
| G52E | 0.1 | S138E | 0.1 | G196K | 0.1 | T141Y | 0 |
| G52T | 0.1 | V139S | 0.1 | G196C | 0.1 | T141D | 0 |
| M72D | 0.1 | V139Q | 0.1 | A203L | 0.1 | T141E | 0 |
| M72E | 0.1 | V139Y | 0.1 | A203T | 0.1 | M193Q | 0 |
| M72T | 0.1 | C140P | 0.1 | F205T | 0.1 | F194Y | 0 |
| Y75Q | 0.1 | C140N | 0.1 | S206H | 0.1 | G195T | 0 |
| Y75D | 0.1 | T141V | 0.1 | T207D | 0.1 | G196R | 0 |
| Y75E | 0.1 | T141G | 0.1 | D209S | 0.1 | S206T | 0 |
| Y75T | 0.1 | T141S | 0.1 | D209Q | 0.1 | D209E | 0 |
| G114Q | 0.1 | P142V | 0.1 | D209T | 0.1 | V211Y | 0 |
| G114Y | 0.1 | P142G | 0.1 | G210D | 0.1 | L216Q | 0 |
| G114D | 0.1 | P142S | 0.1 | V211Q | 0.1 | P222D | 0 |
| G114E | 0.1 | P142Q | 0.1 | L216S | 0.1 | L224T | 0 |
| G114T | 0.1 | P142Y | 0.1 | L216E | 0.1 | L225Y | 0 |
| H115Y | 0.1 | P142D | 0.1 | L216T | 0.1 | Y233V | 0 |
| H115D | 0.1 | P142E | 0.1 | I219Q | 0.1 | Y233G | 0 |
| H115E | 0.1 | P142T | 0.1 | L224E | 0.1 | Y237D | 0 |
| H115T | 0.1 | L153T | 0.1 | Y237Q | 0.1 | L245D | 0 |
| W117Q | 0.1 | M162D | 0.1 | G247T | 0.1 | N250G | 0 |
| W117Y | 0.1 | M162E | 0.1 | P248T | 0.1 | N250S | 0 |
| W117D | 0.1 | M162T | 0.1 | L249E | 0.1 | N250T | 0 |
| W117E | 0.1 | L163R | 0.1 | W251D | 0.1 | S284Y | 0 |
| W117T | 0.1 | L163D | 0.1 | R253Q | 0.1 | D286T | 0 |
| G118S | 0.1 | L163E | 0.1 | F280H | 0.1 | A288E | 0 |
| G118Q | 0.1 | L163T | 0.1 | A282Y | 0.1 |  |  |
| G118Y | 0.1 | N164T | 0.1 | A283T | 0.1 |  |  |
| G118D | 0.1 | K166T | 0.1 | K285T | 0.1 |  |  |
| G119R | 0.1 | L169T | 0.1 | M293Y | 0.1 |  |  |
| G119D | 0.1 | V176T | 0.1 | V43E | 0 |  |  |
